# Toward One Giga Frames per Second — Evolution of *in Situ* Storage Image Sensors

**DOI:** 10.3390/s130404640

**Published:** 2013-04-08

**Authors:** Takeharu G. Etoh, V. T. Son Dao, Tetsuo Yamada, Edoardo Charbon

**Affiliations:** 1 Research Organization of Science and Technology, Ritsumeikan University, Noji-Higashi 1-1-1, Kusatsu 525-8577, Japan; E-Mail: vtsdao@fc.ritsumei.ac.jp; 2 Faculty of Engineering, Tokyo Polytechnic University, Iiyama 1583, Atsugi 243-0297, Japan; E-Mail: yamatetu123@gaea.ocn.ne.jp; 3 Department of Microelectronics, Technische Universiteit, Delft, Mekelweg 4, 2628 CD Delft, The Netherlands; E-Mail: e.charbon@tudelft.nl

**Keywords:** imaging device, high-speed, image sensor, BSI, ISIS, ISAS, PACS 42.79.Pw

## Abstract

The ISIS is an ultra-fast image sensor with in-pixel storage. The evolution of the ISIS in the past and in the near future is reviewed and forecasted. To cover the storage area with a light shield, the conventional frontside illuminated ISIS has a limited fill factor. To achieve higher sensitivity, a BSI ISIS was developed. To avoid direct intrusion of light and migration of signal electrons to the storage area on the frontside, a cross-sectional sensor structure with thick pnpn layers was developed, and named “Tetratified structure”. By folding and looping in-pixel storage CCDs, an image signal accumulation sensor, ISAS, is proposed. The ISAS has a new function, the in-pixel signal accumulation, in addition to the ultra-high-speed imaging. To achieve much higher frame rate, a multi-collection-gate (MCG) BSI image sensor architecture is proposed. The photoreceptive area forms a honeycomb-like shape. Performance of a hexagonal CCD-type MCG BSI sensor is examined by simulations. The highest frame rate is theoretically more than 1Gfps. For the near future, a stacked hybrid CCD/CMOS MCG image sensor seems most promising. The associated problems are discussed. A fine TSV process is the key technology to realize the structure.

## 1. Introduction

Application of high-speed video cameras is expanding to various fields of sciences, including bio-medical sciences and engineering. To meet the ever-growing performance demands for improved sensitivity, frame rate, and pixel count, the image sensors for high-speed imaging have introduced several innovations. Very high sensitivity has been achieved by single-photon imaging technologies [1]. Even in 2000, imaging at 40,500 frames per second (fps) was applied to capturing cavitation bubbles released by a snapping shrimp [2]. The standard frame rate of the camera was 4,500 fps for 256 × 256 pixels, and the sensitivity was enhanced by directly attaching an image intensifier with a micro-channel plate to the image sensor [3]. By increasing the frame rate with partial readout of only 64 × 64 pixels, the motion of the cavitation cloud was successfully captured.

While the highest frame rate of video cameras is continuously being renewed, there are still many important phenomena that cannot be imaged even with the most advanced high-speed video cameras. For example, in biology, microscopic imaging of signal transfer on a nerve cord requires more than 10 Mega fps (frame interval: 100 ns); in scientific instrumentation, fluorescence life-time microscopy (FLIM) requires a temporal resolution of 100 ps [4,5]. The paper reviews the evolution of the ultra-high-speed image sensors in the past, and forecasts future evolutions.

Since the development of a digital-recording high-speed video camera in 1991, Etoh and his colleagues have been updating the highest frame rate of high-speed video cameras: 4,500 frames per second (fps) in 1991 [3], one million fps (1 Mfps) in 2001 [6], and 16 Mfps in 2011 [7]. The color version with 300 kpixel was developed in 2006 [8]. The latest version has achieved 16.7 Mfps for 300 kpixels [9]. The past evolution has been documented in the series of their previous review papers [10–13]. New image sensor structures have been developed to achieve much higher frame rate and higher sensitivity, and to introduce additional useful functions [14]. A simulation study shows that it is possible to achieve one Giga fps (1 Gfps) with silicon semiconductor technology [15].

Image signals generated in an image sensor with a global shutter are read out of the sensor through the following process:

[a. Generation of an electron-hole pair]
➔ [b. Travel of the photoelectron to a collection element in each pixel]
➔ [c. Transfer of a packet of the photo-electrons, *i.e.*, an image signal, to a neighboring storage area simultaneously at all pixels]
➔ [d. Transfer of the image signal to a readout circuit on the periphery of the image sensor chip]
➔ [e. Readout of the image signals to a buffer memory outside the chip]

The delay of image capturing is associated with the signal transfer process. For example, the first photo-chemical reaction in human eyes completes in less than one hundred femtoseconds. However, the subsequent signal transfer process to the brain takes more than 1 ms, and the final image recognition takes about 100 ms. To compensate for the delay, some insects have *in situ* signal processors in their eyes, and even some dinosaurs were equipped with a local signal processor in their loins. The development history of high-speed video cameras has been making the signal recording devices closer to the signal generation site.

Conventional high-speed video cameras with continuous readout increase the frame rate by utilizing the parallel and partial readout [e. from the image sensors to the outside memory] with the increased number of readout wires [3].

The *in situ* storage image sensor, ISIS, has a local signal storage area with more than 100 memory elements attached to each pixel. During image-capturing, image signals are stored in the *in situ* storage without being read out. The frame interval, the inverse of the frame rate, can be decreased down to [c. the transfer time of an image signal to the *in situ* storage. ] The ISIS chip achieved 1 Mfps [6].

However, the fill factor of the front-side illuminated ISIS was only about 15% due to the light shield covering the *in situ* storage area. To increase the fill factor to nearly 100%, a backside illuminated ISIS (BSI ISIS) was developed. To prevent direct intrusion of incident light and migration of generated photoelectrons into the memory on the front side, a BSI sensor structure consisting of pnpn layers was developed [16]. The frame rate was also drastically increased to 16 Mfps for 165 kpixels by additional wiring on the front side without decrease of the fill factor and violation of pixel uniformity [7].

The transfer of collected photoelectrons to a neighboring storage area takes much longer time than the travel time of photoelectrons to a collection element. Therefore, an image sensor with multiple collection gates placed in a circular geometry in the center of each pixel can achieve a much higher frame rate by collecting generated photoelectrons at one of the in-pixel collection gates and by transferring a signal charge packet from the collection gate to the attached *in situ* storage during collection of photoelectrons at other collection gates. The multi-collection-gate image sensor can reduce the frame interval down to [b. the time for a photoelectron to travel to one of the in-pixel collection elements] [15]. The travelling time can be reduced to less than 1 ns. Therefore, the multi-collection-gate image sensor can achieve theoretically 1 Gfps.

If the signals of a sequence of images are recorded [a. exactly at their generation sites], the ultimate ultra-high-speed imaging can be achieved. Innovative technologies in this category have been proposed [17–19]. Frame intervals of several picoseconds [18] to hundreds of femtoseconds [19] have been achieved. However, silicon image sensors have the significant advantage of providing compact and user-friendly imaging systems.

One of the important additional functions introduced by the authors is in-pixel image signal accumulation. In image capturing of repetitive phenomena under very weak incident light, the S/N ratio can be improved by summing up image signals obtained by repeated capturing. The ISIS chip with folded and looped in-situ CCD storage provides a practical ultra-high-speed image sensor with the in-pixel signal accumulation. For its internal structure, the sensor was called “the image signal accumulation sensor” or ISAS [14]. An ISIS with the CCD memory and the CMOS readout has been reported [20,21]. A pure CMOS version with pixel-based recording has also been developed, and is now a product [22,23]. The sensor has the storage areas attached to each pixel on the periphery of the chip.

## 2. ISIS

### 2.1. ISIS with Slanted Linear CCD Storage

Kosonocky developed the CCD ISIS for the first time [24]. However, the Series-Parallel-Series (SPS) CCD was used for the *in situ* storage, which was difficult to fabricate due to the complexity, resulting in a very low yield rate. Lazovsky developed a CCD ISIS with linear in-situ CCD storage, which achieved 100 Mfps [25]. However, the fill factor, the pixel count and the total number of frames were only 1%, 64 × 64, and 16, which are far below users’ requirements. Therefore, both of them were employed by a very limited number of the users.

**Figure 1.**
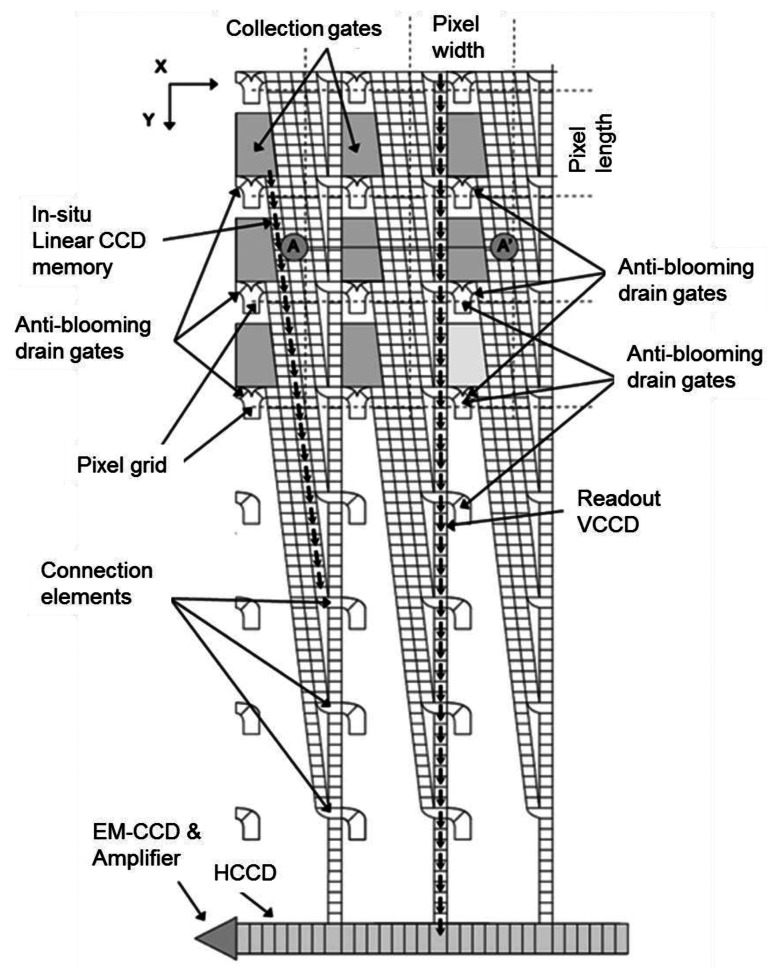
Plane structure of ISIS with slanted linear storage CCDs [7,16].

The first practical CCD ISIS was developed by Etoh *et al.* [6]. Figure 1 shows the ISIS with slanted linear storage CCDs. The collection gates in the figure were the photogates of the original frontside-illuminated (FSI) ISIS. An image signal, a charge packet, generated in a photogate is transferred along a memory CCD, extending linearly in a slightly slanted direction to the pixel grid.

During the image capturing operation, the image signals are continuously transferred downward on the linear storage CCD, and drained out of the sensor from the drain attached at the end. Therefore, the image signals are continuously updated and the latest ones are always stored on the storage CCD. The simple memory structure of the linear CCD maximizes the number of storage elements or minimizes the pixel size for a given number of storage elements. The ISIS achieved 1 Mfps. However, the storage area in each pixel covered with a light shield reduced the fill factor to 15%.

Another problem was very high power consumption, compared with CMOS image sensors. Etoh *et al.* predicted the resurrection of the Kosonocky's SPS CCD ISIS [26], since it consumes much less power than ISIS with the slanted linear CCD storage, and can be produced easily by using an existing fine CMOS process. Crooks *et al.* has since made it [21].

### 2.2. BSI ISIS

**Figure 2.**
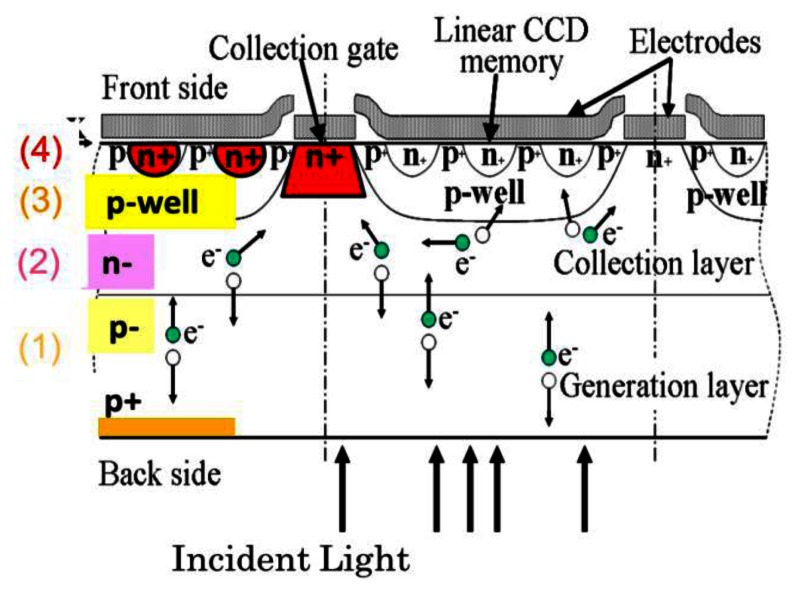
BSI ISIS with a “Tetratified” structure (A-A’ cross section of Figure 1) [7,16].

To improve fill factor, a BSI ISIS structure was developed as shown in Figure 2. To prevent direct intrusion of incident light into the storage area on the front side, the thickness of the sensor was increased to more than 30 μm. For the 30 μm thickness, 0.1% of the 700 nm incident light still reaches the front side. Technology to produce a thicker and low-concentration epi-layer with fewer defects is awaited.

To avoid migration of the generated electrons to the storage area of each pixel, a p-well embracing the n^+^ CCD storage channels is formed in the n^−^ epi-layer grown on a p^−^ epi-layer. The structure with n^−^/p^−^ double epi-layers reduces the backside bias voltage to deplete the photoelectron generation layer; this reduces the electric field and thus dark current during the image capturing operation. The photogate for the FSI ISIS in Figure 1 was modified to the collection gate; generated photoelectrons travelling around the p-well to the collection gate are collected there. The structure consisting of the p^−^/n^−^/p/n^+^ layers was named a “tetratified” BSI image sensor structure, where “tetratified” is an abbreviated expression of “tetra-stratified”.

**Figure 3.**
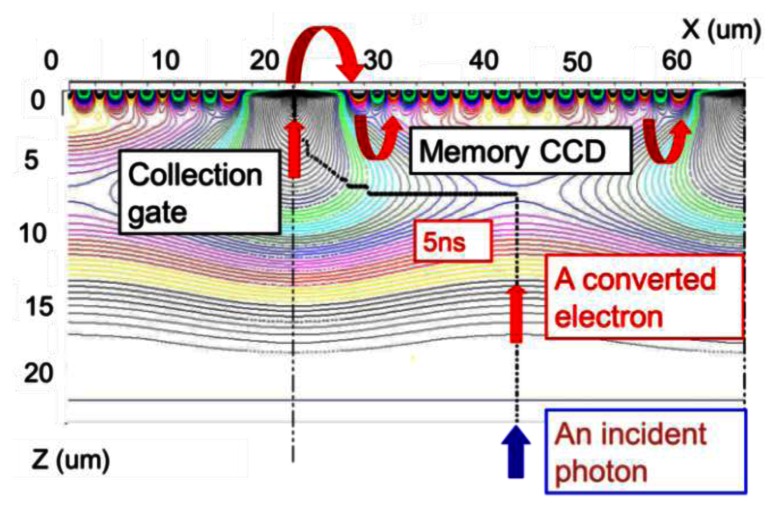
An example ptential profile and an electron path.

Figure 3 shows an example of potential profiles in the BSI ISIS with the slanted linear storage CCDs on the front side.

**Figure 4.**
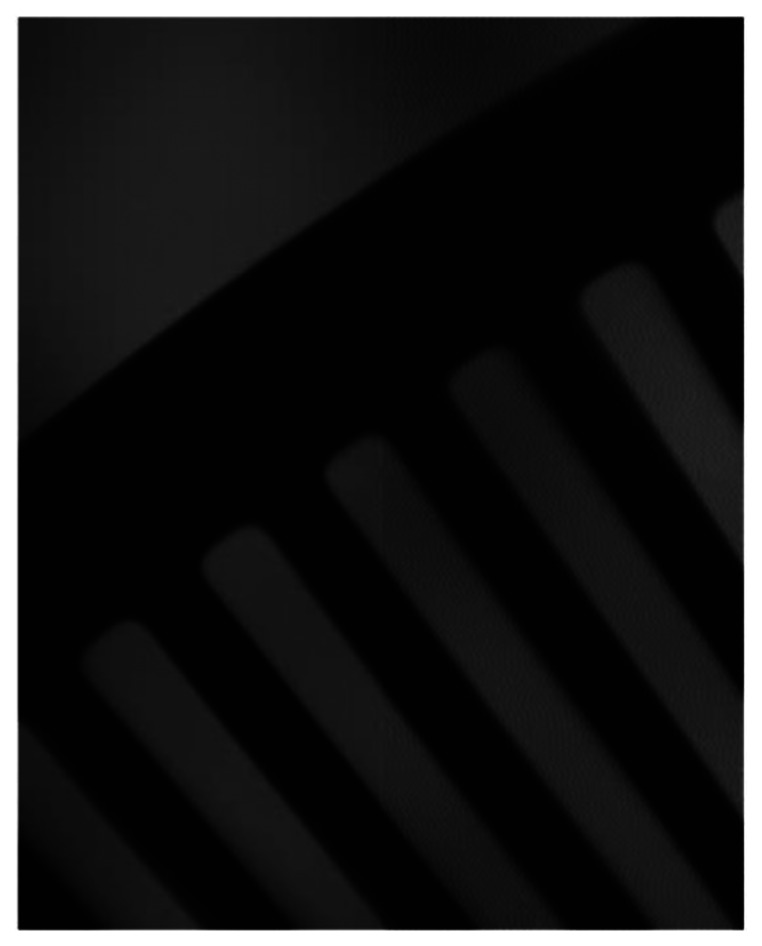
A laser chopper rotating at 6,000 rpm taken by the BSI ISIS at 16 Mfps [7].

The BSI ISIS achieved 16 Mfps for 165 kpixels and very high sensitivity together with the EM-CCD installed in the readout horizontal CCD. An image taken at 16 Mfps is shown in Figure 4.

The sensor is currently being modified for a higher frame rate and a higher pixel count. Arai *et al.* has achieved 16.7 Mfps for 300 kpixels [9]. In the p-well, various functional circuits other than the *in situ* storage can be installed.

A BSI structure partially forming the tetratified structure in a pixel was presented by other scientists and it is known as “quadruple well structure” [27]. Image sensors with various useful functions are being developed, for example, for advanced imaging mas-spectrometry [28].

Silicon-on-insulator (SOI) technology is another option to separate the photo-charge generation layer from the circuit layer [29]. Through a hole in the insulation oxide layer of each pixel, signal holes are transferred to frontside circuits. It is worth comparing the tetratified structure, the quadruple well structure and the SOI separation technologies. A combination of these technologies is also to be considered.

## 3. ISAS

**Figure 5.**
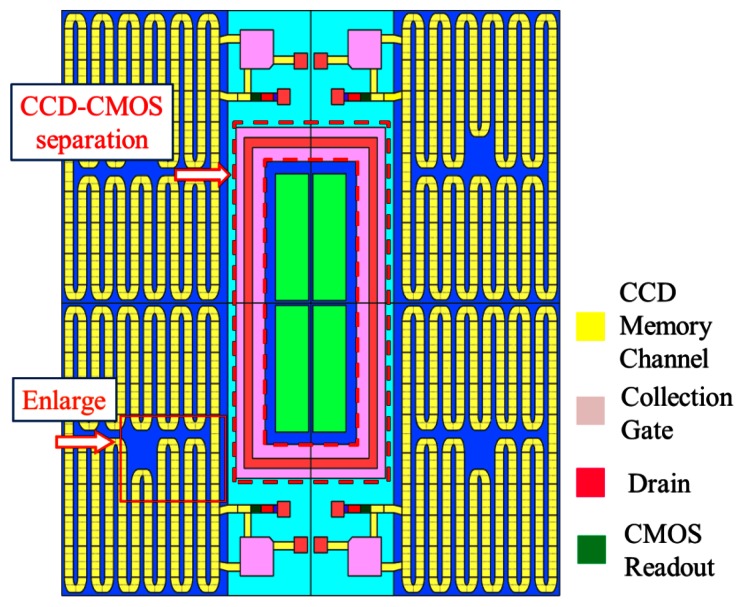
Layout of 2 × 2 pixels in ISAS with folded and looped storage CCDs [14].

The in-pixel image signal accumulation was introduced to the ISIS concept by folding and looping the *in situ* storage CCDs as shown in Figure 5 [14]. The last element of the looped CCD is connected to the first element to achieve charge circulation in multiple image capturing trials. After all the CCD memory elements are filled with the image signals of the first image capturing trial, those of the second trial are automatically added to the stored image signals transferred downward from the storage CCD.

**Figure 6.**
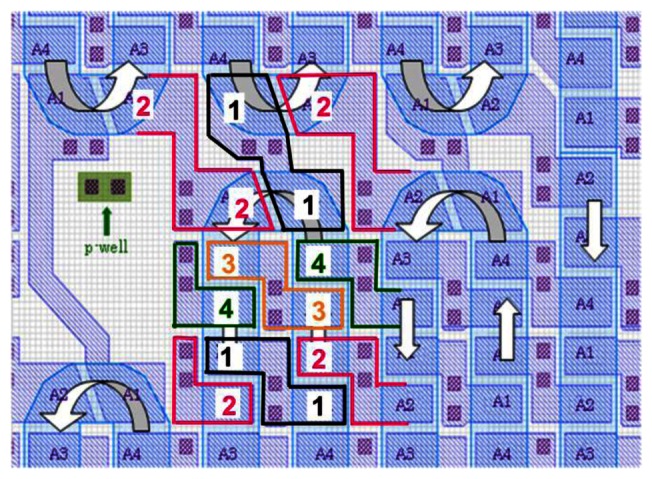
A zoom of the CCD/CMOS ISAS in Figure 5. Z-shaped electrodes enable a folded CCD channel. Those electrodes facing each other across the channel stop automatically change the transfer directions as well [26].

It was difficult to make a multi-folded CCD with fine elements by using the conventional CCD technology with double poly-silicon electrodes. The current CMOS process provides a single silicon electrode layer with the spaces down to 0.1 μm, which is narrow enough to transfer charge packets on a CCD channel with sufficiently high transfer efficiency. As shown in Figure 6, the Z-shaped electrodes nicely change the transfer direction [24].

The ISAS was invented to meet the requirements of scientists in the field of pulse neutron radiography. The flight speed of a neutron is nearly proportional to its energy; interaction of the neutron to materials is dependent of the energy and the atoms or their states in the specimen through which the neutron passes. Therefore, the spatial distributions of the atoms in the specimen can be detected by measuring the attenuation and the arriving time of neutrons to the pixels [30]. The ISAS provides time-resolved imaging with sufficient time resolution for this purpose.

The time-of-flight (TOF) imaging by the ISAS can also be applied to mass spectrometry, called in this case “Imaging TOF MS”. Since the sensor is BSI, electron or ion bombardment is applicable. The electron energy 8 keV for the direct bombardment is recommended in [31].

## 4. Hybrid CCD/CMOS ISIS

A hybrid CCD/CMOS ISIS was also developed. Each pixel is equipped with a linear storage CCD and a CMOS readout circuit [20]. The main advantage of the structure is that it enables both ultra-high-speed imaging and high-speed readout. An issue inherent to the structure is the thickness of the insulation layer. CCDs can operate effectively for an oxide thickness of more than 20 nm, desirably 50 nm. The oxide thickness of the current CMOS process is less than 8 nm, thus a better compromise should be sought. The latest version of the hybrid CCD/CMOS ISIS achieved 2 Mfps for 700 kpixels and 180 consecutive frames [21].

## 5. CMOS Image Sensor with Pixel-Based Recording

In the past, many CMOS based ISIS type sensors were experimentally designed and fabricated. El-Desouki *et al.* listed up high-speed imagers to show superiority of their CMOS *in situ* memory image sensor [32,33]. However, it was difficult to yield successful products for practical applications. The main reason lies in the working principle of CMOS image sensors: an image signal generated in a photodiode is amplified to compensate for kTC noise and leakage before being transferred to memory. Since the signal at the memory element is much larger than the original one, larger memory elements are required: this results in a smaller number of the *in situ* storage elements, *i.e.*, the number of the consecutive frames.

To solve the problem, Kleinfelder *et al.* employed PIP capacitors, which comprise a polysilicon electrode, an insulation layer, and another polysilicon electrode, for *in situ* frame storage [34,35]. Oxidation of the surface of the bottom polysilicon electrode provides a very thin dielectric insulation layer, and, thus, high capacitance. Akahane, Sugawa *et al.* combined the PIP capacitor with a conventional MOS capacitor to create a compact analogue storage unit with higher capacitance to develop wide dynamic range image sensors [36].

Tochigi *et al.* employed the capacitor for the pixel-based storage, and finally succeeded to develop a practical ultra-high-speed CMOS image sensor [22,23]. Another advantage of this implementation is the light shield performance, since the storage area is separated from the photo-receptive area and placed in the peripheral light-shielded area of the chip. The CMOS switching and multi-parallel signal transfer wires on each pixel column made the separation possible. The sensor is equipped with a current source in each pixel to eliminate shading due to attenuation of the driving power at the inner pixels.

In the next stage, it is expected that stacking technology is applied to the *in situ* storage image sensors with the storage on the different tier connected to the sensor tier with CMOS amplification circuit in each pixel. An early example of the stacked CMOS ISIS is presented in [37].

## 6. Multi-Collection-Gate Image Sensor

### 6.1. Macro-Pixel Operation to Multi-Collection-Gate Sensors

The frame rate can be quadrupled by grouping pixels of an ISIS type sensor to macro-pixels, each with independently operated 2 × 2 pixels, and by operating them in turn. Usually, the travelling time of a photoelectron to a collection site in a pixel is shorter than the transfer time of the collected image signal charge to the *in situ* storage. If the transfer time is less than three times the travelling time, an image signal collected at one of four pixels is transferred completely to the storage area during collection of image signals at the other three pixels in turn.

The disadvantage is that, during the collection of photoelectrons in one pixel, photons incident to the other three pixels are lost, and therefore, the fill factor of the macro pixel becomes less than 25%. Namely, all four pixels in a group pixel receive practically the same number of incident photons in a frame interval. To temporally resolve the signals to four pixels, photoelectrons generated in one pixel must be collected, and those generated in the rest of three pixels must be drained out of the sensor.

**Figure 7.**
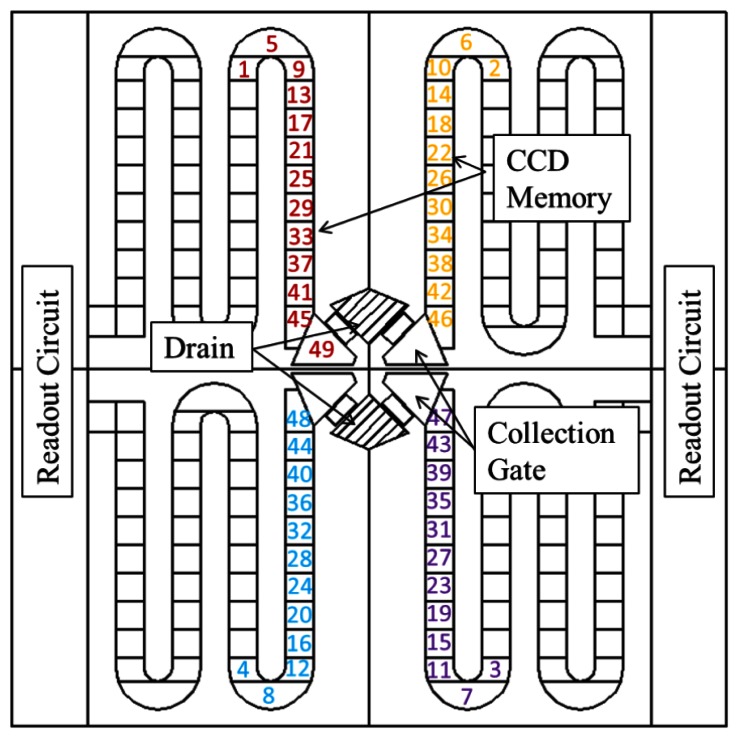
A tetragonal CCD multi-collection-gate image sensor.

The tetratified BSI structure solves the problem. A conceptual model of the pixel is shown in Figure 7. Four collection gates of the pixels are centered, to each of which an *in situ* storage CCD is attached. They are protected from the migration of signal electrons by a p-well built with a couple of the masks similar to those shown in Figure 11 later. The p-well has an n-type hole at the center, and is thicker at the periphery of each pixel and thinner toward the center, which creates a potential gradient to the center and nicely accelerates electrons toward the center, as “an electronic microlens”. Electrons passing through the central hole to the front side are collected by a collection gate where a higher voltage is applied. The fill factor is thus 100%.

### 6.2. Honeycomb Multi-Collection-Gate Image Sensors

**Figure 8.**
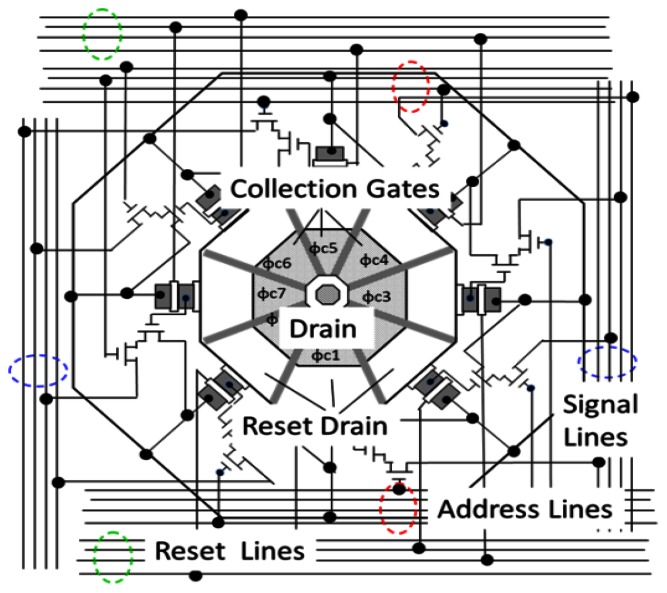
An octagonal CMOS multi-collection-gate image sensor.

**Figure 9.**
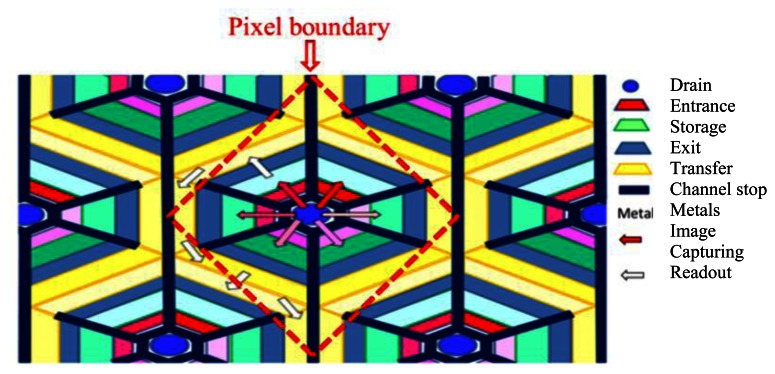
A distorted hexagonal CCD multi-collection-gate BSI image sensor.

When the number of collection gates in a pixel is eight or six, the shape of the pixel becomes octagonal or hexagonal as shown in Figure 8 or 9. When the imaging area is filled with pixels, the photo-receptive area is formed like a honeycomb. Figure 8 shows a model of the frontside circuit of a stacked hybrid CCD/CMOS architecture image sensor with octagonal pixels. Figure 9 shows a pure CCD sensor with hexagonal pixels. To make the pixel grid square, the hexagons are distorted.

The BSI image sensor with the structure is named “Multi-Collection-Gate Backside-Illuminated image sensor (MCG BSI image sensor)”.

### 6.3. Preliminary Simulation

**Figure 10.**
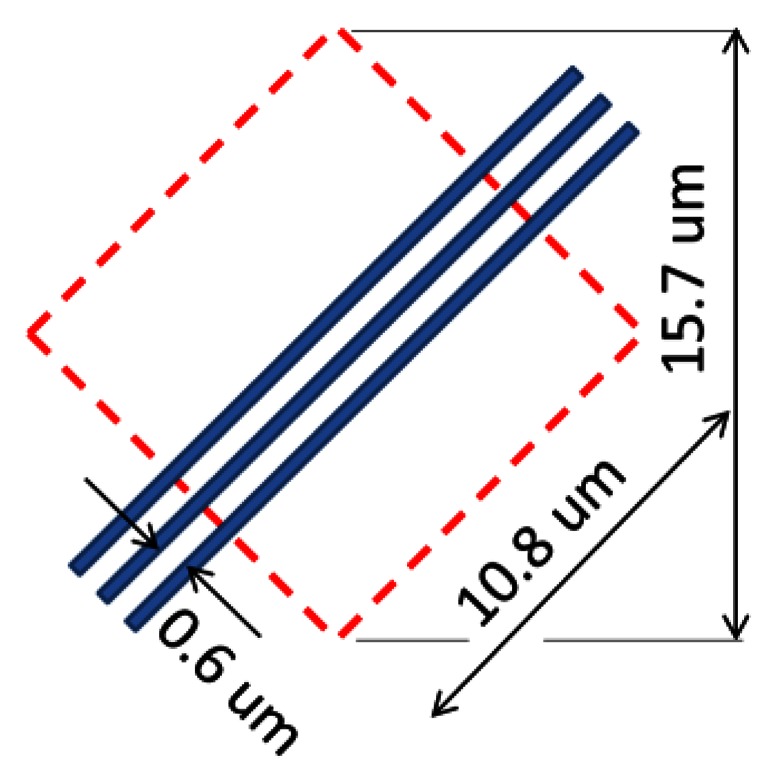
Pixel size and metal wiring direction and pitch.

**Figure 11.**
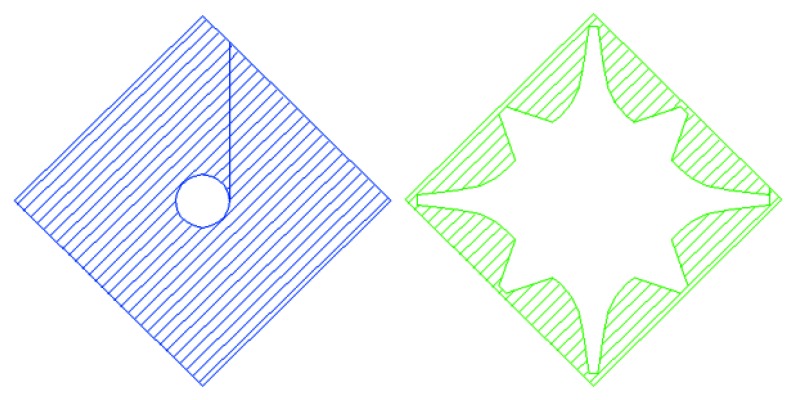
Masks for the p-well creating an electronic microlens (Negative).

The core technology of the MCG BSI image sensor is selective collection of signal electrons by one specified collection gate, which is also the most difficult part in the design. To prove the validity of the technology, preliminary simulations were conducted for a hexagonal MCG BSI image sensor shown in Figures 9, 10 and 11. By covering the frontside circuit with a deep p-well made with a couple of masks shown in Figure 11, signal electrons generated by incident photons to the square area shown in Figure 10 are collected by one of the collection gates. A collection gate consists of an entrance gate and a storage gate, followed by an exit barrier gate and a transfer gate. The structure of all the gates is the buried CCD. Direction of the second metal wires is parallel to that of the pixel boundary as shown in Figure 10. The pixel configuration is suitable for the interlace imaging. The chip can be rotated 45 degrees when it is designed or mounted on a camera, if necessary.

The size of a pixel is 10.8 μm. Eighteen metal wires are necessary to deliver voltages to drive a pixel. For 0.13 μm process, the pitch of the second metal wires was fixed at 0.6 μm with some allowance, which determined the size of the pixel (0.6 μm × 18 = 10.8 μm). The thickness of the chip is 33 μm, which consists of an 11 μm n-epi and a 22 μm p-epi layers.

During the image capturing operation, voltages of all storage gates are kept at a higher level. When the voltage of one of the entrance gates is raised, keeping the others at the low level, the signal electrons are collected by the collection gate through the raised entrance gate.

The voltage of the exit barrier gate is kept at a middle level. When a voltage of a storage gate is lowered after a burst image capturing of six signals, a signal charge stored in the storage gate overflows over the exit gate to one of the transfer gate, and is read out downward through the transfer gates around the exit gates.

**Figure 12.**
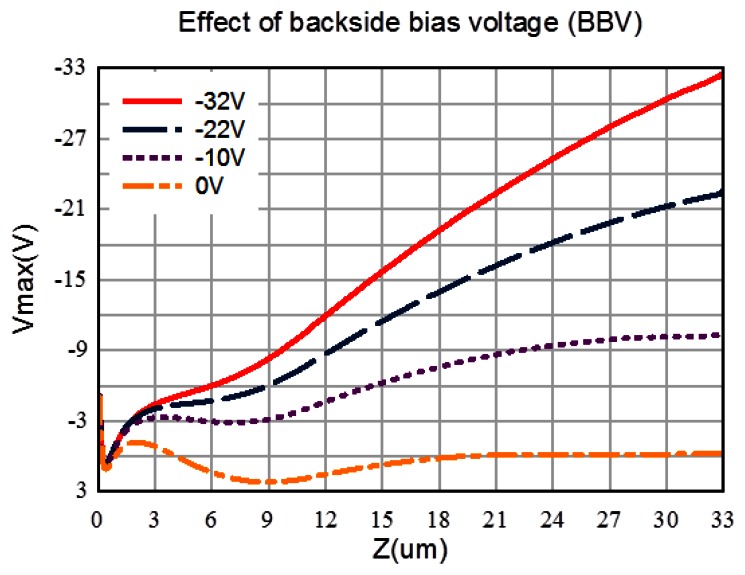
Potential profiles in z-direction at the center *vs.* backside voltage.

Figure 12 shows potentials in the depth (z) direction at the center of a pixel. If the backside voltage is lower than −22 V, the signal electrons safely travel to the frontside; if it is higher than −10 V, the signal electrons can no longer directly go to a collection gate, being blocked by a potential dip in the path, which implies possibility of a vertical drain electronic shutter.

**Figure 13.**
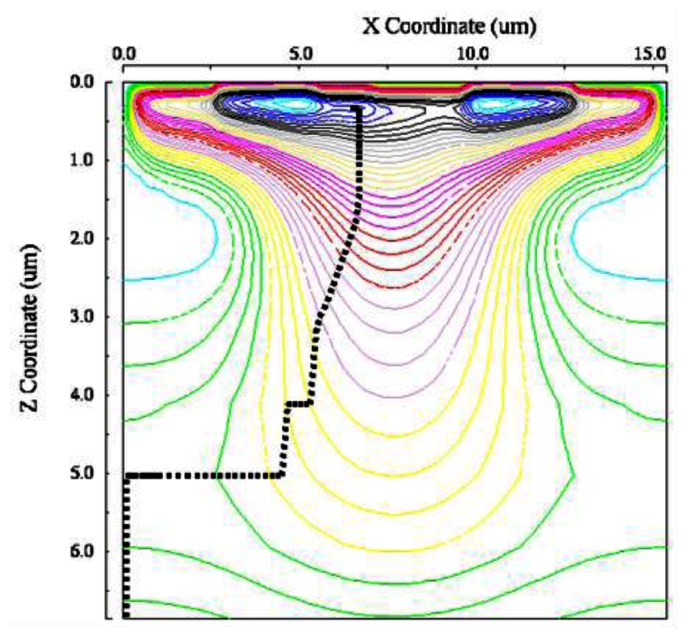
Selective collection of an electron generated at the “left” corner by the “left” collection gate (0 < z < 6.9 μm).

**Figure 14.**
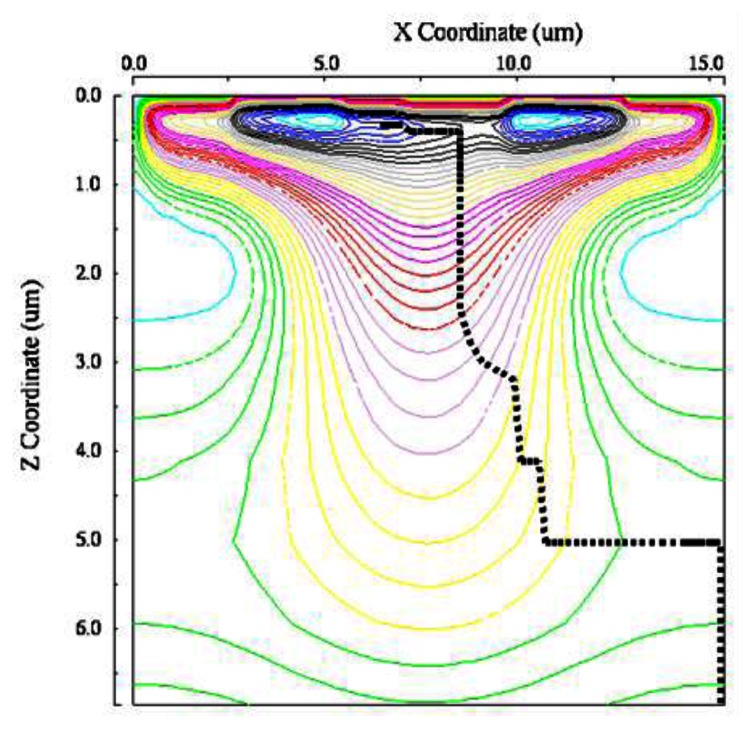
Selective collection of an electron generated at the “right” corner by the “left” collection gate (0 < z < 6.9 μm).

**Figure 15.**
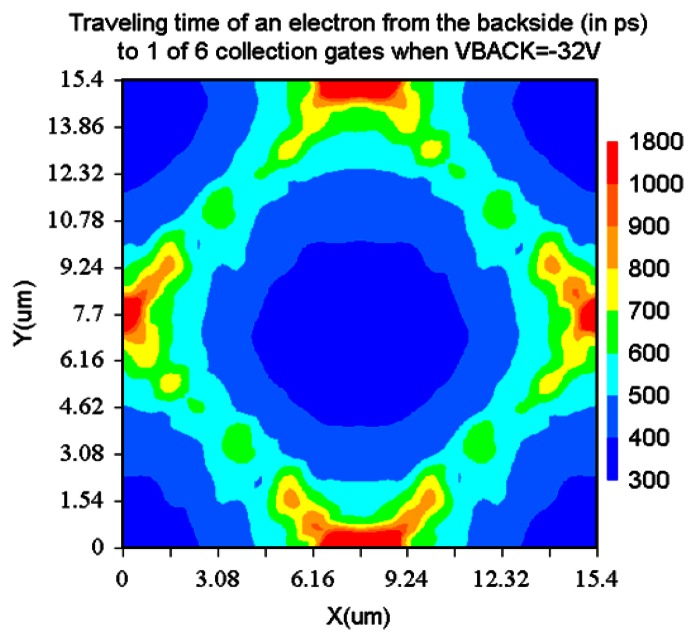
Traveling time of an electron from a point of the backside to 1 of 6 collection gates (ps) (Backside voltage −32 V).

**Figure 16.**
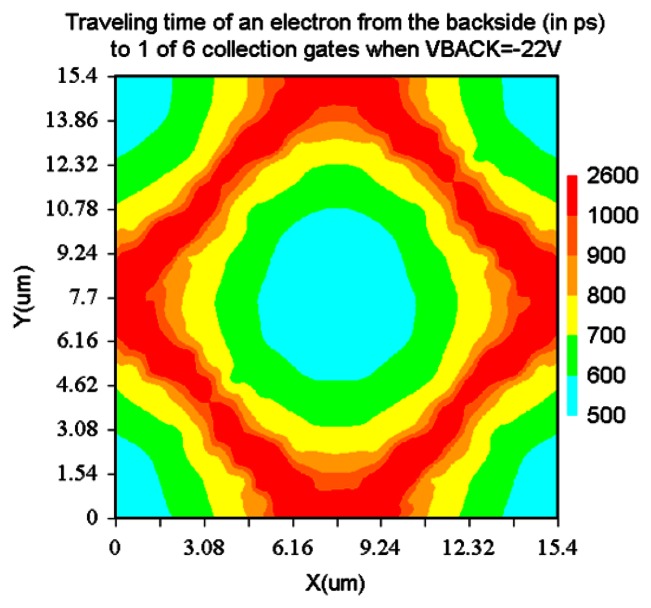
Traveling time of an electron from a point of the backside to 1 of 6 collection gates (ps) (Backside voltage −22 V).

Figures 13 and 14 show paths of an electron generated at the left or the right corner of a pixel. The voltage of the left-side entrance gate is at the high level. Figures 15 and 16 show the travelling time of an electron from a point of the backside to a collection gate. It depends on the backside voltage. For the backside voltage −32 V, more than 95% of signal electrons reach the collection gate in less than 1ns, and, thus, practically, 1Gfps is achievable. However, an electron generated at the left or right corner of the pixel shown in Figure 13 or 14 takes more than 1.5 ns. The time can be reduced to less than 500 ps by collecting the incident light to the central area of a pixel with an optical microlens.

For the backside voltage −22 V, the microlens is necessary to achieve 1Gfps. However, the dark current may significantly reduce for the less electric field from the backside to the frontside.

## 7. Evolving Designs

### 7.1. Image Signal Amplification

When a very small number of incident photons are available, which is inherent to ultra-high-speed imaging, very high sensitivity with efficient signal amplification technologies, such as electron bombardment [31], EM-CCD [38], and SPAD [39], must be incorporated. These technologies are listed up and summarized elsewhere as single-photon imaging technologies [1].

### 7.2. Stacked Driver Chips

Even if the pixel is itself fast, distributing control signals to it in a massive array is a challenge and may hinder further frame rate increase. It is difficult to operate multi-collection-gate image sensors at 1 Gfps by using a driving system made with commercially available electronic components. It is challenging to distribute 1 GHz clocks over a massive pixel array with little skew. Thus, it is necessary to develop a dedicated driver system and to optimize it. The drivers should be placed as close as possible to each pixel or each pixel block. Only a stacked sensor structure makes it possible, whereas local oscillators synchronized among each other will provide high-speed clock.

Stacking technology is being steadily improved [40]. An image sensor with three stacked chips has already been successfully fabricated in a trial to introduce higher functions to image sensor chips [41]. We believe that this technology will be mature for our applications in the near future.

### 7.3. Continuous Digital Recording

One digital memory chip can store image signals for a sufficient number of frames for practical ultra-high-speed imaging. Therefore, a stacked multi-collection-image sensor architecture will enable ultra-high-speed continuous-recording, which overcomes the limited number of frames, the major shortcoming of pixel-based recording image sensors, such as an ISIS. It takes a long time to read out image signals of many frames to the outside of the stacked image sensor. To keep the image signals for a long time on the image sensor chip, they must be stored in digital format.

A crucial factor to realize the continuous digital recording at a very high frame rates is the throughput rate from an imaging chip to the attached memory chip. The throughput rate is defined by a product of the dynamic range (ADC resolution), the pixel count (spatial resolution), and the frame rate (temporal resolution), and is limited by the product of the throughput rate per TSV and the total number of the TSVs.

Since a very small number of photons are available, a low-resolution ADC can be employed. A pixel-based 4-bit ADC operates at a sample rate of more than one hundred Mega samples per second (100 Msps). An octagonal multi-collection-gate image sensor multiplies the throughput eight times. Therefore, it may be possible to digitize image signals captured at more than 0.1 Gsps and transfer them at about 1 Gsps to a stacked memory chip.

Consequently, the highest technical barrier is the shrinkage of TSVs and contact points between chips to increase the density. Their fine process is eagerly awaited to realize continuous imaging at 1 Gfps.

Timing control is another crucial problem. An advanced ring oscillator technology makes it possible to keep timing errors within 100 ps (<1 ns), independently of voltage and temperature shifts via continuous compensation, as in phase-locked loop.

## 8. Concluding Remarks

**Figure 17.**
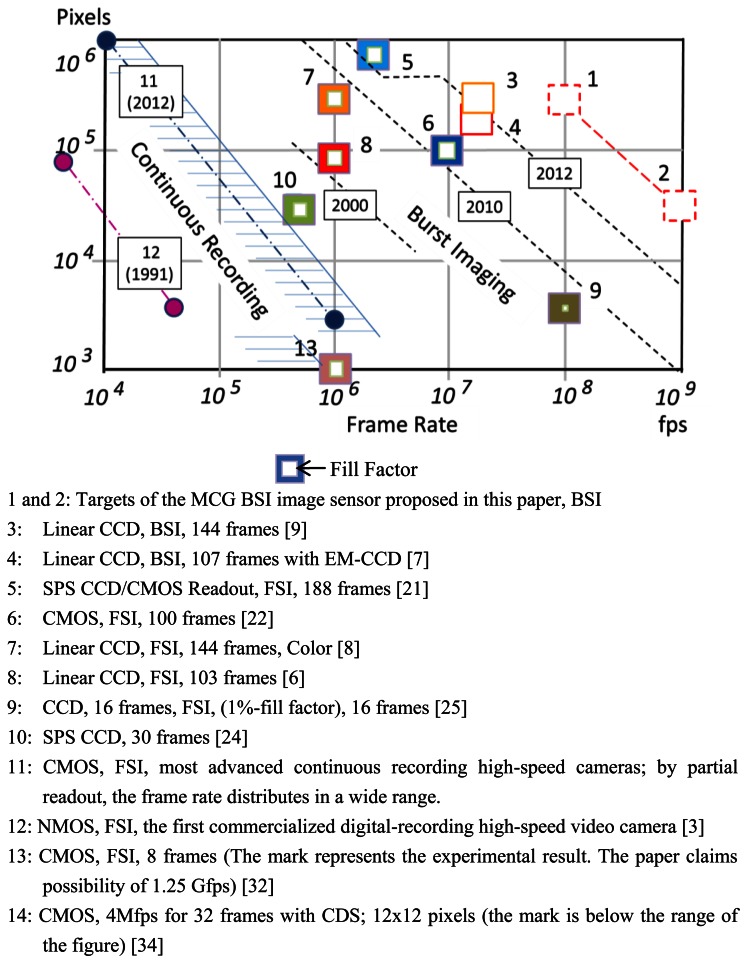
Evolution of ultra-high-speed video cameras: Frame rate *vs.* Pixel count.

The ISIS is the ultra-fast image sensor with in-pixel storage. The evolution of the ISIS in the past and in the near future is reviewed and forecasted. The process is depicted in Figure 17.

To cover the storage area with a light shield, the conventional frontside illuminated ISIS has a limited fill factor. To achieve higher sensitivity, a BSI ISIS was developed. To avoid direct intrusion of light and migration of signal electrons to the storage area on the frontside, a cross-sectional sensor structure with thick pnpn layers was developed, and named “Tetratified structure”. The structure contributed to increase the frame rate as well as the sensitivity, with metal wiring on the frontside with higher freedom. The highest frame rate of the existing ISIS with this structure is 16.7 Mfps for 300 kpixels.

A different way to avoid the direct intrusion of light and the electron migration to the *in situ* storage is developed by Tochigi *et al.* The pixel-based storage is placed on the periphery covered with an efficient light shield.

Another problem associated with the ISIS with the slanted linear CCD was very high power consumption, compared with CMOS image sensors. Etoh *et al.* suggested that the Kosonocky's SPS CCD ISIS consumes much less power, and can be easily produced by using an existing fine CMOS process. Crooks *et al.* has revived the Kosonocky's model.

By folding and looping in-pixel storage CCDs, an image signal accumulation sensor, ISAS, is proposed. The ISAS has a new function, the in-pixel signal accumulation, in addition to the ultra-high-speed imaging.

To achieve much higher frame rate, a multi-collection-gate (MCG) BSI image sensor architecture is proposed. Around the center of each pixel, plural of collection gates are placed and collect image signals in turn. A signal charge collected by a collection gate is transferred to a neighboring storage area during signal collection by other collection gates. The photoreceptive area forms a honeycomb-like shape. Performance of a hexagonal CCD-type MCG BSI sensor is examined by simulations. The highest frame rate is theoretically more than 1 Gfps.

For the near future, a stacked hybrid CCD/CMOS MCG image sensor seems most promising. The associated problems are discussed. A fine TSV process is the key technology to realize the structure.
